# Adipose and skin distribution of African trypanosomes in natural animal infections

**DOI:** 10.1186/s13071-024-06277-7

**Published:** 2024-05-11

**Authors:** Cynthia Mmalebna Amisigo, Gloria Amegatcher, Jack D. Sunter, Theresa Manful Gwira

**Affiliations:** 1 West African Centre for Cell Biology of Infectious Pathogens, University of Ghana, Accra, Ghana; 2 Department of Biochemistry, Cell and Molecular Biology, University of Ghana, Accra, Ghana; 3 Department of Biological and Medical Sciences, Oxford Brookes University, Oxford, UK

**Keywords:** Trypanosome, Tissue tropism, Skin, Adipose, Livestock

## Abstract

**Background:**

Animal African trypanosomiasis, which is caused by different species of African trypanosomes, is a deadly disease in livestock. Although African trypanosomes are often described as blood-borne parasites, there have been recent reappraisals of the ability of these parasites to reside in a wide range of tissues. However, the majority of those studies were conducted on non-natural hosts infected with only one species of trypanosome, and it is unclear whether a similar phenomenon occurs during natural animal infections, where multiple species of these parasites may be present.

**Methods:**

The infective trypanosome species in the blood and other tissues (adipose and skin) of a natural host (cows, goats and sheep) were determined using a polymerase chain reaction-based diagnostic.

**Results:**

The animals were found to harbour multiple species of trypanosomes. Different patterns of distribution were observed within the host tissues; for instance, in some animals, the blood was positive for the DNA of one species of trypanosome and the skin and adipose were positive for the DNA of another species. Moreover, the rate of detection of trypanosome DNA was highest for skin adipose and lowest for the blood.

**Conclusions:**

The findings reported here emphasise the complexity of trypanosome infections in a natural setting, and may indicate different tissue tropisms between the different parasite species. The results also highlight the need to include adipose and skin tissues in future diagnostic and treatment strategies.

**Graphical Abstract:**

**Figure:**
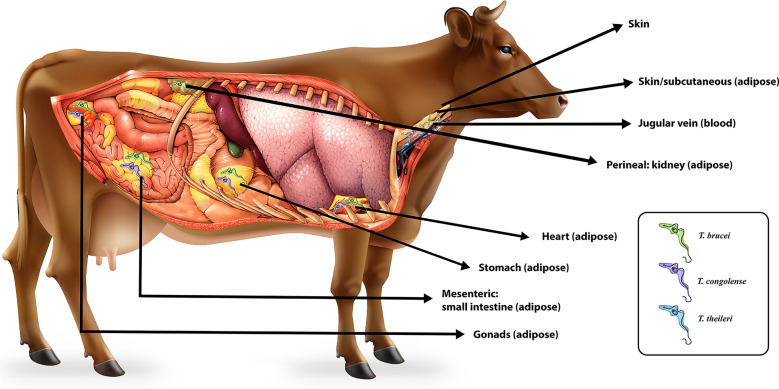

**Supplementary Information:**

The online version contains supplementary material available at 10.1186/s13071-024-06277-7.

## Background

Animal African trypanosomiasis (AAT) is a disease that affects both domestic and wild animals in sub-Saharan Africa. Animal trypanosome infection is characterized by fever, listlessness, emaciation, hair loss, anaemia, paralysis and eventually death if no treatment is administered [1]. In livestock farming, this important infectious disease leads to serious economic losses, e.g. an estimated US $1–1.2 billion loss in cattle production annually [2]. AAT is caused by several trypanosome species, including *Trypanosoma brucei, Trypanosoma congolense* and *Trypanosoma vivax* [3]*.* The disease normally occurs in areas inhabited by the biological vectors of trypanosomes (tsetse flies *Glossina* spp.), although some species of *Trypanosoma* can be mechanically and sexually transmitted [3].

Although numerous investigations, some of which date back to the early 1900s, demonstrated that *T. brucei* has the ability to invade a wide range of tissues and organs of various types of livestock, *T. vivax* and *T. congolense* were considered to be restricted to the vasculature; however, studies have shown that these parasites can also be detected in the adrenal cortex and dermis of experimentally infected animals [4–10]. Despite these findings, molecular studies of experimental *T. brucei* infections in mice have generally focused exclusively on the parasites that are found in blood, although there has been a recent rekindling of interest in the biology of those that can reside in other tissues [11–13]. However, most data on trypanosomes resident in adipose and skin tissues are from mouse models, and the few experiments that did use a natural host only examined the presence of the parasites in the skin and subcutaneous adipose tissue [11, 14–17]. The distribution of adipose-resident parasites in other adipose deposits, such as gonadal, mesenteric, heart or perirenal adipose, has not been determined in natural hosts during natural animal infections.

In this study, we identified and characterized the trypanosome species in the blood, skin and adipose tissue of livestock to further understand the tissue distribution of these parasites in natural infections.

## Methods

### Study area and sample collection

The study was carried out at two government-approved abattoirs [Accra Abattoir and Johnny’s Food and Meat Complex (JFAMCO)] located in the Greater Accra region of Ghana. The animals that were sampled appeared to be healthy and were cleared by the veterinary officer for slaughter. Animals were sampled whenever they were available for slaughter, regardless of the season. Sex, breed and possible origin were recorded (Table 1), and the samples were taken post-mortem. A total of 242 samples were obtained from 35 animals comprising 20 goats, 10 cows and five sheep. The samples included peripheral blood from the jugular vein, skin biopsies from the neck region and fat/adipose tissue from the gonads, heart, intestine, skin, kidney and/or stomach. Blood was taken immediately after slaughter, and the other tissues were collected, at most, at 15 min after slaughter. For the blood samples, 5 ml of blood was collected in EDTA vacutainers, while the other tissues (1.6–10.5 g) were thoroughly washed with water to remove any residual blood before being placed in absolute alcohol for 1–2 h. All samples were transported on ice to the laboratory for DNA extraction.

**Table 1:** Characteristics and detection rate of trypanosomes in livestock slaughtered at Accra Abattoir and Johnny’s Food and Meat Complex, Greater Accra region, Ghana

Livestock breed (no. of animals) and livestock identifiers	Possible origin of livestock	Total no. of positive samples	Detection rate per livestock breed sample	Overall detection rate per sample
Cows		26		10.8%
N’dama (8); C1A-C6A, C8A, C10A	Northern and southern regions	22	32.5%	9.1
Sanger (2); C7A, C10A	Northern and southern regions	4	5.9%	1.7
Sheep		19		7.9%
West African long-legged (5); S1A, S2A, S1J-S3J	Not determined	19	54.3%	7.9%
Goats		74		30.6%
West African dwarf (6); G1A-G4J	Southern region	11	7.9%	4.6%
Sahelian long-legged (14); G5J-G18J	Northern and upper regions	63	45.3%	26.0%

Total no. of tissue samples (total = 242 for all 35 animals sampled) used to calculate the overall detection rate; total no. of tissue samples per type of animal (cows = 68, sheep = 35, and goats = 139) used to calculate the livestock breed detection rate

### DNA isolation

Genomic DNA was extracted from the blood (200 µl) and other tissues (25 mg) using DNeasy Blood & Tissue Kit (QIAGEN, Germany), following the manufacturer’s protocol with slight modifications. For adipose tissue, the samples were incubated overnight at 56 °C after addition of the tissue lysis buffer ATL (QIAGEN) and 20 µl of proteinase K. Extracted DNA samples (5–70 ng/µl) were stored at − 20 °C.

### Identification of trypanosome species using nested polymerase chain reaction

The first round of the polymerase chain reaction (PCR) amplification was performed in 25-μl reaction volumes containing 5 μl of DNA and 20 μl of master mix, comprising 1× Mango *Taq* buffer with 100 mM dNTPs, 1.5 mM MgCl_2_, 10 μM outer primers and 12.5 μl water. The nested primers bind to the intergenic region of the alpha–beta tubulin gene cluster, which is able to differentiate between different trypanosome species based on the length of the amplified region and the primer sequence [18]. The PCR amplification for the first run was done using 30 cycles, with an initial denaturation at 94 °C for 4 min, followed by denaturation at 94 °C for 40 s, primer annealing at 61 °C for 40 s, polymerization at 72 °C for 45 s and final extension at 72 °C for 5 min. PCR amplification for the nested step was done using the same reaction volumes, with 5 μl of the first PCR product, inner primers and the same cycling conditions as the first PCR reaction. The positive control samples contained DNA of *T. congolense* and *T. brucei* and the negative control contained no DNA. The nested PCR products were loaded on a 1.5% agarose gel and the trypanosomes were identified based on the band sizes of the PCR products. The expected band sizes by nested PCR are as follows: 424 base pairs (bp) for *T. brucei*, 456 bp for *T. congolense*, 586 bp for *T. vivax*, and 646 bp for *Trypanosoma theileri.* The DNA markers used included Directload™ Wide Range DNA Marker (D7058; Sigma-Aldrich) for samples C1A-C7A, G3A, G4A, G4J, G5J, G7J, G9J, G10J, G17J, G18J, S1A, S2A; Quick-Load^®^ Purple 100-bp DNA Ladder (no. N0551; NEB) for sample C8A; and Quick-Load^®^ Purple 1-kb plus DNA ladder (no. N0550; NEB) for samples C9A-C10A, G1A, G2A, G3J, G6J, G8J, G11J-G16J and S1J-S3J.

Further identification and characterization were performed by Sanger sequencing, with the focus on trypanosome-positive samples. Gel extraction was performed on selected positive samples using the QIAquick gel extraction kit (QIAGEN). PCR products were validated via Sanger sequencing using a sequencing primer (5′ AGCCGCTCTGGTTGCCGC 3′) that targets the alpha–beta tubulin intergenic region. Prior to the Basic Local Alignment Search Tool analysis, the obtained sequences were trimmed at both ends by eliminating bases with a quality score below 20. The Basic Local Alignment Search Tool analysis of the sequences was conducted using TriTrypDB, and the sequences that had the highest similarity scores were selected.

### Data analysis

Fisher’s exact test was used to compare the detection rates of trypanosome infections between blood and other tissues. A *P*-value of < 0.05 was considered significant.

## Results

### Different patterns of tissue distribution

**Fig. 1:**
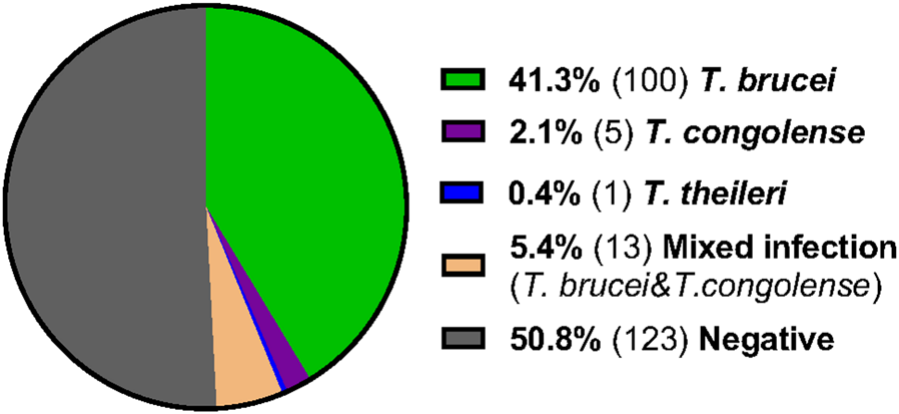
Incidence of infection of identified trypanosome species. Total no. of tissue samples tested was 242. Total no. of positives and negatives are indicated in parentheses

The presence of trypanosome DNA in naturally infected blood, skin and adipose tissue of the animals (comprising 20 goats, 10 cows and five sheep) was determined using nested PCR and confirmed via Sanger sequencing (Additional file 1: Table S1 and Additional file 2). Trypanosome DNA was detected in 49.2% (119 out of 242) of the samples. *T. brucei* was the predominant species, present in 100 samples, while *T. congolense* DNA was exclusively detected in five samples, and *T. theileri* DNA was only detected in one sample. Thirteen of the tissue samples contained DNA of both *T. brucei* and *T. congolense*, and accounted for 5.4% of the samples (Fig. 1).

**Fig. 2:**
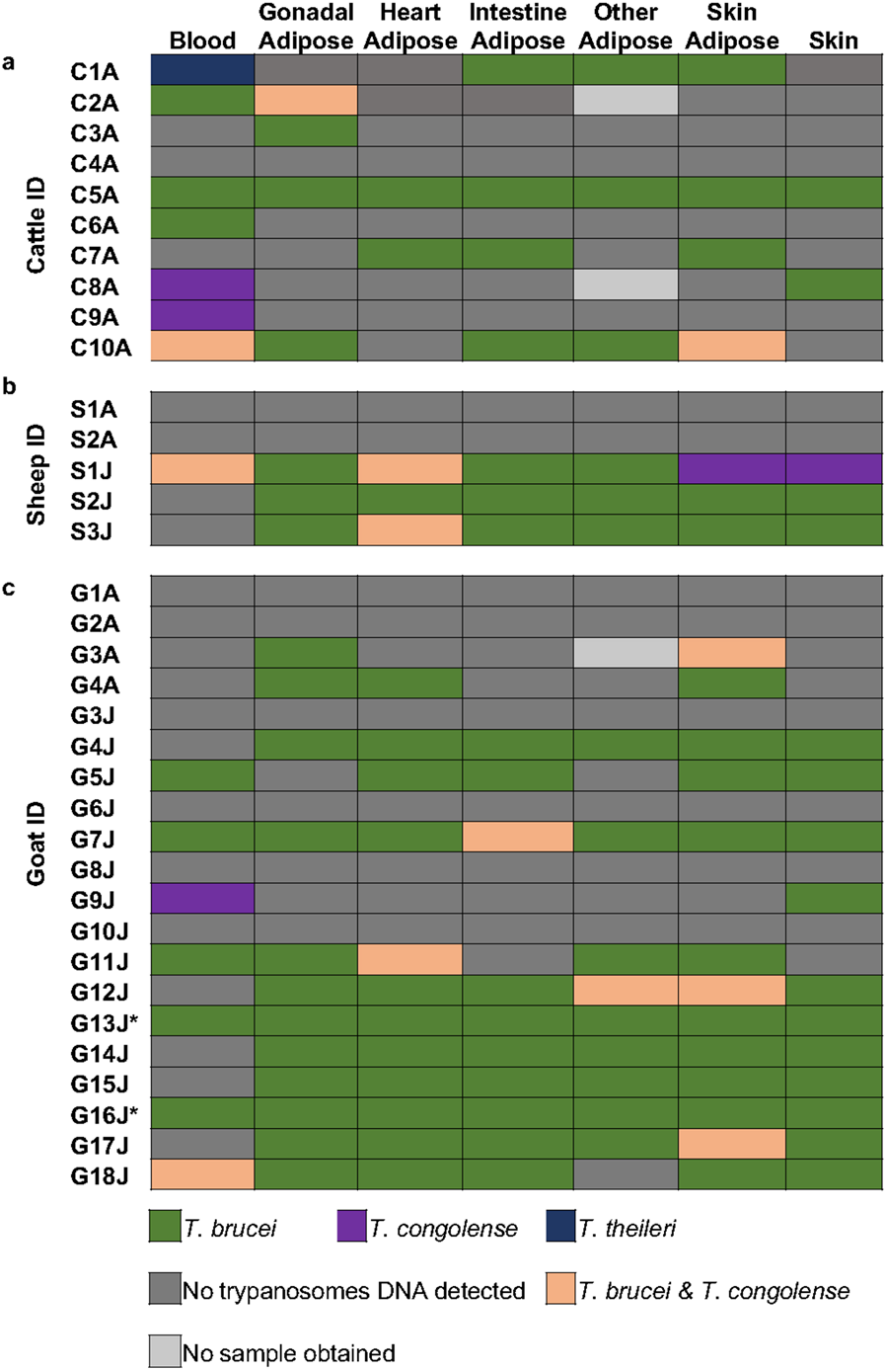
Trypanosome species distribution in tissues of **a** cattle, **b** sheep and **c** goats. An asterisk indicates that the* Other adipose* tissue was obtained from the kidney; all* Other adipose* tissue was obtained from the stomach

Trypanosome DNA was detected in at least one sample from the vast majority of the animals, and was only absent from the samples of one cow, two sheep and six goats (Fig. 2). AT JFAMCO, the majority of the sampled goats were of the Sahelian long-legged breed (G5J-G18J), which is primarily raised in the northern and upper regions of Ghana [19]. These regions have a high trypanosome prevalence and tsetse fly density [20], and the positive detection rate for trypanosome DNA was much higher for the samples from these goats [Sahelian long-legged; 45.3% (63 out of 139 goat samples)] than from those of the West African dwarf goats (G1A-G4J) [7.9% (11 out of 139 goat samples)], which are primarily raised in the south of the country (Table 1; Fig. 2c).

Interestingly, there was no consistent pattern of detected trypanosome DNA across the different samples. Some animals were negative for trypanosome DNA in both blood and other tissues, some were negative for blood but positive for other tissue, while others were positive for blood but negative for other tissues (Fig. 2; Additional file 1: Figs. S1–S3). Data obtained from the nested PCR diagnosis and the Sanger sequencing are summarized in Additional file 1: Table S1 and Additional file 2. Overall, species identification according to the nested PCR matched that of the sequencing data, with only one mismatch. The PCR-based diagnostic predicted the presence of *T. theileri*, while the sequencing data showed the presence of *T. congolense* genomic DNA, which indicates that nested PCR diagnosis is a good diagnostic tool for this type of application with regards to its sensitivity and specificity.

### Trypanosome prevalence between tissues

The number of trypanosome-positive samples was compared across the various tissues (Table 2). The highest number of positive samples (16.8%—20 out of 119) was for skin adipose, and the lowest for blood (12.6%—15 out of 119). One of the highest trypanosome DNA detection rates (16.0%, 19 out of 119) was for gonadal adipose, while the rate was 14.3% (17 out of 119) for both the heart and intestinal adipose. Trypanosome DNA was detected in 12.6% (15 out of 119) and 13.4% (16 out of 119) of the other adipose and skin samples, respectively (Table 2). However, the number of trypanosome-positive samples did not significantly differ between between the blood and other tissues (Fisher’s exact test).

**Table 2:** Detection rate of trypanosome species’ DNA identified from livestock tissues

Tissues	Total no. of positive samples	Detection rate	Statistical analysis (Fisher’s exact test)
Blood	15	12.6%	
Gonadal adipose	19	16.0%	*P* = 0.4734
Heart adipose	17	14.3%	*P* = 0.8106
Intestinal adipose	17	14.3%	*P* = 0.8106
Other adipose tissue	15	12.6%	*P* > 0.9999
Skin adipose	20	16.8%	*P* = 0.3391
Skin	16	13.4%	*P* > 0.9999

Total no. of positive samples for all tissues = 119

## Discussion

Recent studies have highlighted the presence of trypanosome reservoirs in the dermis and adipose tissue of mice and humans, which has significant implications for the transmission, diagnosis and treatment of trypanosomiasis [11, 16, 21]. However, a clear picture of the distribution of these parasites in livestock is currently lacking. Our study indicates that different trypanosome species can be found in skin and various adipose deposits, in addition to the blood, within the same animal. This hints at a potential difference in tissue preference between trypanosome species, which may reduce competition within the host, as has been suggested for the different developmental stages of trypanosomes in their tsetse fly vectors [22]. However, the location of different parasites in different tissues in the present study may have just been a reflection of stochastic changes in the ongoing infections in these animals, and further work is necessary to determine the root causes of our observations.

We did not detect *T. vivax* infections in the present study, in contrast to previous reports from Ghana, and many other parts of West Africa, where this species is considered the most important cause of AAT in cattle [18, 23–26]. The low detection rate of *T. congolense* and lack of detection for *T. vivax* may have been due to the intravascular nature of these parasites, which reduces the chances of detecting them in blood samples from the jugular vein, and/or indicate that they do not accumulate in adipose tissue, although *T. vivax* has been found in adipose tissue in mice [27, 28]. 

Among the three pathogenic trypanosomes of livestock—*T. brucei*, *T. vivax* and *T. congolense*—*T. brucei* is the least pathogenic, and animals infected with it exhibit fewer pathological signs than animals infected with the two other species [29, 30]. The high positivity rate reported here for *T. brucei* DNA in comparison to DNA of the two other species may, therefore, have been due to the fact that animals with a *T. brucei* infection appeared to be sufficiently healthy for slaughter, although they had a latent infection or were asymptomatic, and may also have been animal reservoirs, as seen in cases of *Trypanosoma brucei gambiense* infection in humans [31].

African trypanosomes are usually described as hemoflagellates, and thus are expected to be detected in the blood. However, in the present study, the detection rate was lowest in the blood, and in some animals parasite DNA could be detected in other tissues even when it was absent from the blood. These findings are supported by those of Capewell et al. [11], who detected trypanosomes in the skin and subcutaneous adipose tissue of mice even in the absence of blood parasitemia. It should be noted, however, that the approach that we employed could not differentiate DNA in the vasculature of a tissue from that in the fluid. In addition, as current diagnostic techniques for trypanosomes generally rely on venous blood samples [32], a lower detection rate for the blood is of potential concern, as it suggests that the techniques used in screening programmes could lead to misdiagnosis, especially for animal infections that are frequently characterized by low blood parasitemia.

The presence of *T. brucei* and *T. congolense* in the skin supports the findings of Dwinger et al. [33] and Capewell et al. [11], who suggested that these parasites could establish and multiply there following a blood meal by the vector. A more recent study, by Ekloh et al. [34], also detected the DNA of *T. brucei*, *T. congolense* and *T. theileri* in the skin of a naturally infected cow, even after its treatment with diminazene aceturate. In addition, Capewell et al. [11] were able to demonstrate that skin not only serves a reservoir but may also serve as an active site for transmission of the parasites.

The evidence presented here, showing that the DNA of trypanosomes can be detected in different adipose tissue of their natural hosts, supports findings from mouse studies [16, 28, 35]. The results also indicate that adipose tissue may be an important reservoir for these parasites in their natural hosts. Future studies need to use quantitative approaches to determine the number of trypanosomes within the different tissues of infected animals to understand their relative contribution to the parasites’ maintenance and transmission.

## Conclusions

In summary, our data demonstrate that, during a natural infection, trypanosome DNA can be readily detected in the skin and adipose tissue of various organs, even when it is not present in the blood. These results highlights the need to examine additional tissues, such as in the skin and adipose tissue, when performing diagnostic tests for trypanosomes. Moreover, the DNA of different trypanosome species may be distributed among different tissues within the same animal, which suggests that trypanosomes may differ with respect to their tissue tropisms.

### Supplementary Information

**Additional file 1:** **Figure S1.** Molecular characterization of* Trypanosoma* species in cattle tissues, using nested PCR targeting part of the tubulin gene cluster. **Figure S2.** Molecular characterization of* Trypanosoma* species in sheep tissues, using nested PCR targeting part of the tubulin gene cluster. **Figure S3.** Molecular characterization of* Trypanosoma* species in goats tissues, using nested PCR targeting part of the tubulin gene cluster. **Table S1.** PCR confirmation via Sanger sequencing.

**Additional file 2.** Sanger sequencing data.

## Data Availability

All data generated or analysed during this study are included in this published article and its additional files.

## Abbreviations

BLAST: Basic Local Alignment Search Tool
PCR: Polymerase Chain Reaction
