# Supplementary material for: Adipose and skin distribution of African trypanosomes in natural animal infections
Source: Parasit Vectors. 2024 May 11;17:215. doi: 10.1186/s13071-024-06277-7 (PMC11088761; doi:10.1186/s13071-024-06277-7)
Supplement: Supplementary file 1 — Additional file 1: Figure S1. Molecular characterization of Trypanosoma species in cattle tissues, using nested PCR targeting part of the tubulin gene cluster. Figure S2. Molecular characterization of Trypanosoma species in sheep tissues, using nested PCR targeting part of the tubulin gene cluster. Figure S3. Molecular characterization of Trypanosoma species in goats tissues, using nested PCR targeting part of the tubulin gene cluster. Table S1. PCR confirmation via Sanger sequencing. [file 13071_2024_6277_MOESM1_ESM.pptx]

## Slide 1
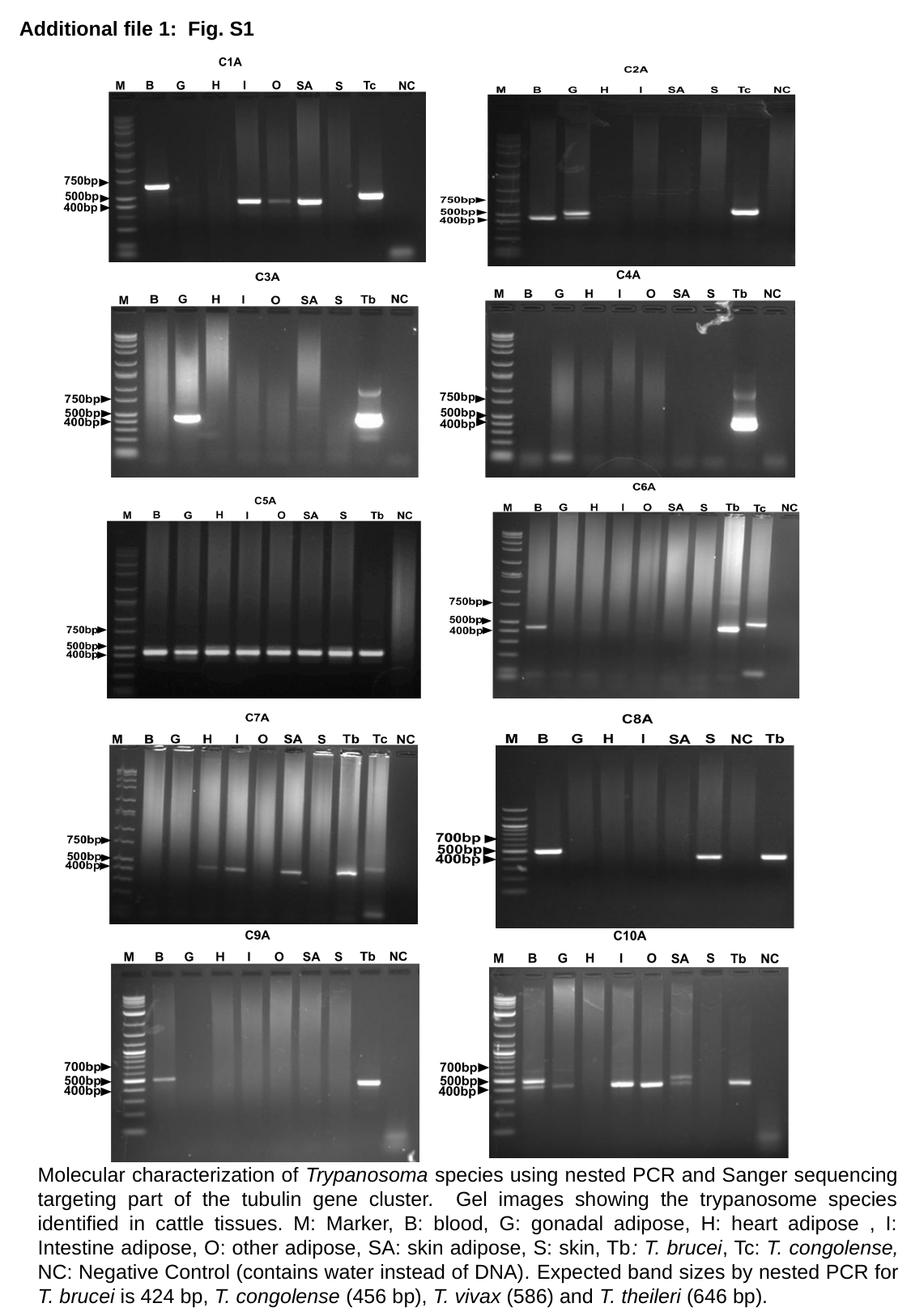

Additional file 1: Fig. S1
Molecular characterization of Trypanosoma species using nested PCR and Sanger sequencing targeting part of the tubulin gene cluster. Gel images showing the trypanosome species identified in cattle tissues. M: Marker, B: blood, G: gonadal adipose, H: heart adipose , I: Intestine adipose, O: other adipose, SA: skin adipose, S: skin, Tb: T. brucei, Tc: T. congolense, NC: Negative Control (contains water instead of DNA). Expected band sizes by nested PCR for T. brucei is 424 bp, T. congolense (456 bp), T. vivax (586) and T. theileri (646 bp).

## Slide 2
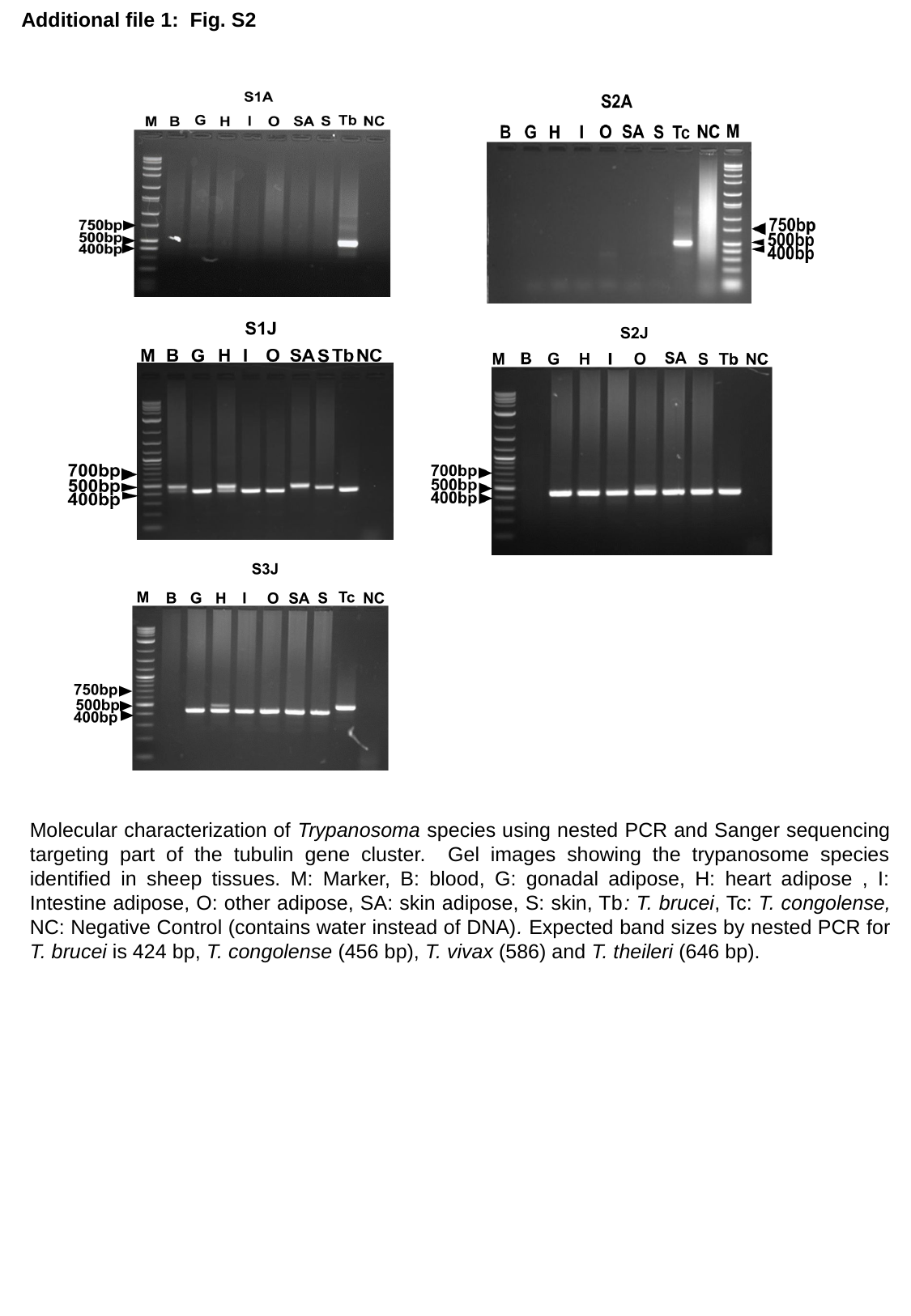

Additional file 1: Fig. S2
Molecular characterization of Trypanosoma species using nested PCR and Sanger sequencing targeting part of the tubulin gene cluster. Gel images showing the trypanosome species identified in sheep tissues. M: Marker, B: blood, G: gonadal adipose, H: heart adipose , I: Intestine adipose, O: other adipose, SA: skin adipose, S: skin, Tb: T. brucei, Tc: T. congolense, NC: Negative Control (contains water instead of DNA). Expected band sizes by nested PCR for T. brucei is 424 bp, T. congolense (456 bp), T. vivax (586) and T. theileri (646 bp).

## Slide 3
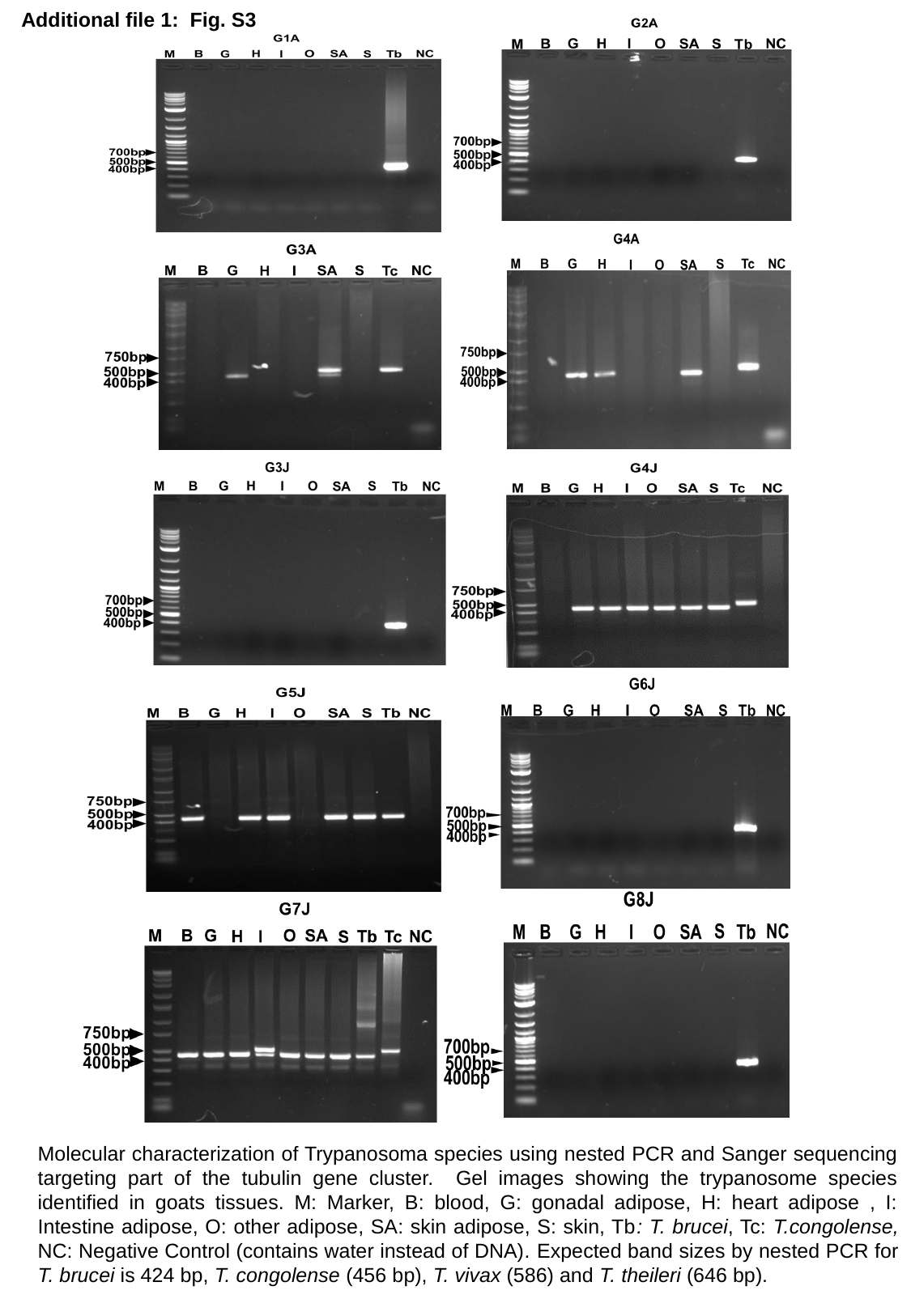

Additional file 1: Fig. S3
Molecular characterization of Trypanosoma species using nested PCR and Sanger sequencing targeting part of the tubulin gene cluster. Gel images showing the trypanosome species identified in goats tissues. M: Marker, B: blood, G: gonadal adipose, H: heart adipose , I: Intestine adipose, O: other adipose, SA: skin adipose, S: skin, Tb: T. brucei, Tc: T.congolense, NC: Negative Control (contains water instead of DNA). Expected band sizes by nested PCR for T. brucei is 424 bp, T. congolense (456 bp), T. vivax (586) and T. theileri (646 bp).

## Slide 4
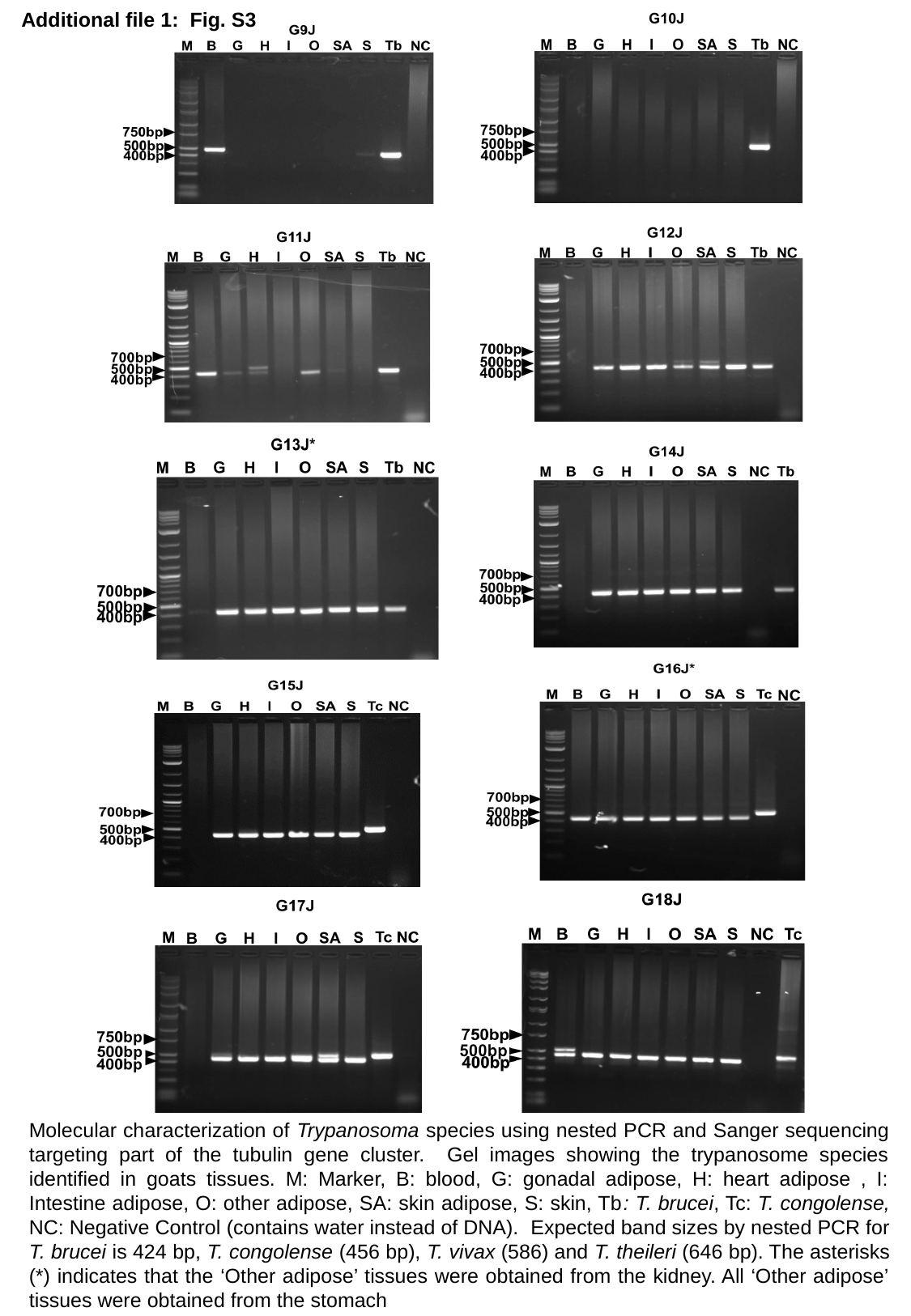

Additional file 1: Fig. S3
Molecular characterization of Trypanosoma species using nested PCR and Sanger sequencing targeting part of the tubulin gene cluster. Gel images showing the trypanosome species identified in goats tissues. M: Marker, B: blood, G: gonadal adipose, H: heart adipose , I: Intestine adipose, O: other adipose, SA: skin adipose, S: skin, Tb: T. brucei, Tc: T. congolense, NC: Negative Control (contains water instead of DNA). Expected band sizes by nested PCR for T. brucei is 424 bp, T. congolense (456 bp), T. vivax (586) and T. theileri (646 bp). The asterisks (*) indicates that the ‘Other adipose’ tissues were obtained from the kidney. All ‘Other adipose’ tissues were obtained from the stomach

## Slide 5
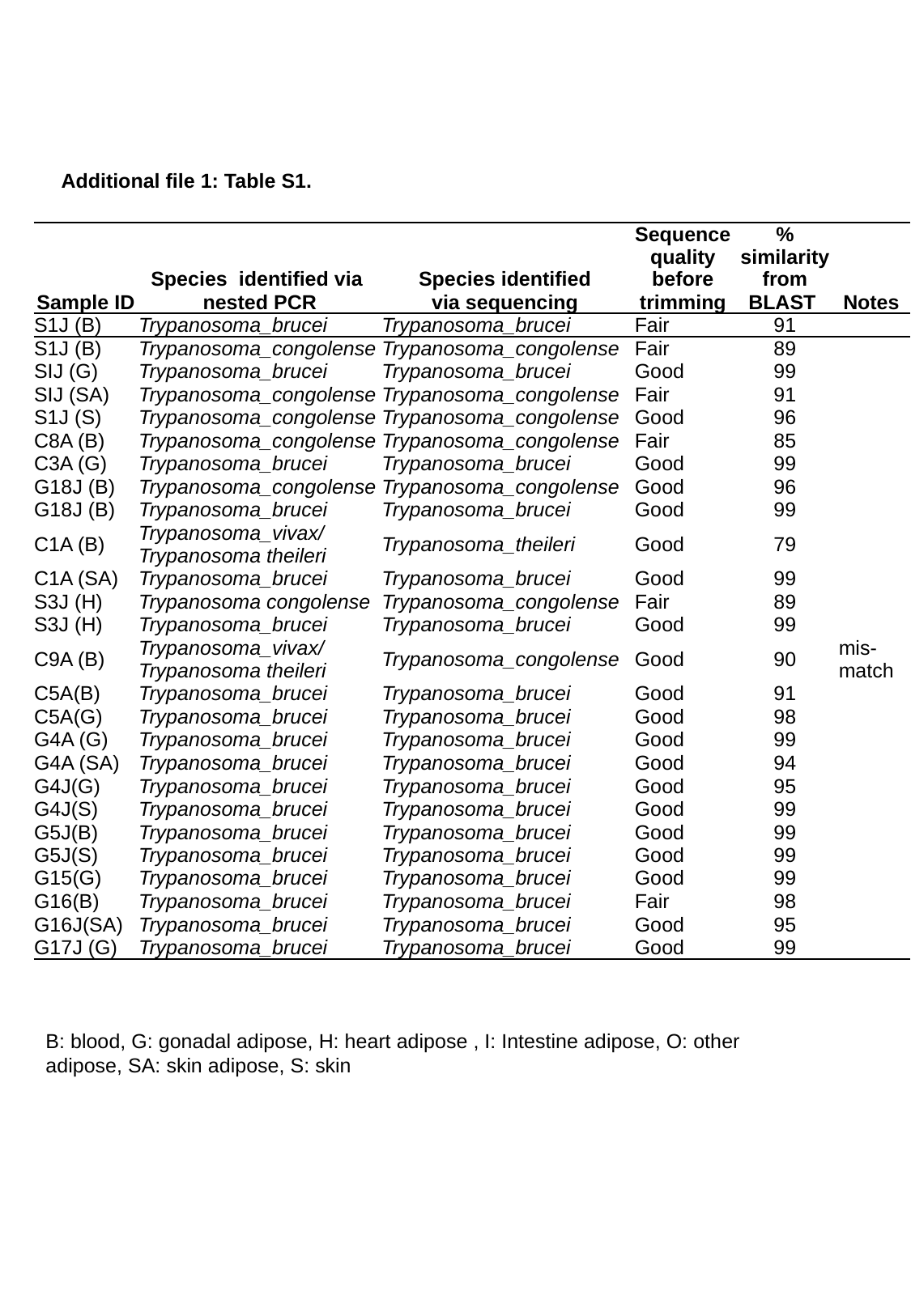

Additional file 1: Table S1.
| Sample ID | Species identified via nested PCR | Species identified via sequencing | Sequence quality before trimming | % similarity from BLAST | Notes |
| --- | --- | --- | --- | --- | --- |
| S1J (B) | Trypanosoma\_brucei | Trypanosoma\_brucei | Fair | 91 | |
| S1J (B) | Trypanosoma\_congolense | Trypanosoma\_congolense | Fair | 89 | |
| SIJ (G) | Trypanosoma\_brucei | Trypanosoma\_brucei | Good | 99 | |
| SIJ (SA) | Trypanosoma\_congolense | Trypanosoma\_congolense | Fair | 91 | |
| S1J (S) | Trypanosoma\_congolense | Trypanosoma\_congolense | Good | 96 | |
| C8A (B) | Trypanosoma\_congolense | Trypanosoma\_congolense | Fair | 85 | |
| C3A (G) | Trypanosoma\_brucei | Trypanosoma\_brucei | Good | 99 | |
| G18J (B) | Trypanosoma\_congolense | Trypanosoma\_congolense | Good | 96 | |
| G18J (B) | Trypanosoma\_brucei | Trypanosoma\_brucei | Good | 99 | |
| C1A (B) | Trypanosoma\_vivax/ Trypanosoma theileri | Trypanosoma\_theileri | Good | 79 | |
| C1A (SA) | Trypanosoma\_brucei | Trypanosoma\_brucei | Good | 99 | |
| S3J (H) | Trypanosoma congolense | Trypanosoma\_congolense | Fair | 89 | |
| S3J (H) | Trypanosoma\_brucei | Trypanosoma\_brucei | Good | 99 | |
| C9A (B) | Trypanosoma\_vivax/ Trypanosoma theileri | Trypanosoma\_congolense | Good | 90 | mis-match |
| C5A(B) | Trypanosoma\_brucei | Trypanosoma\_brucei | Good | 91 | |
| C5A(G) | Trypanosoma\_brucei | Trypanosoma\_brucei | Good | 98 | |
| G4A (G) | Trypanosoma\_brucei | Trypanosoma\_brucei | Good | 99 | |
| G4A (SA) | Trypanosoma\_brucei | Trypanosoma\_brucei | Good | 94 | |
| G4J(G) | Trypanosoma\_brucei | Trypanosoma\_brucei | Good | 95 | |
| G4J(S) | Trypanosoma\_brucei | Trypanosoma\_brucei | Good | 99 | |
| G5J(B) | Trypanosoma\_brucei | Trypanosoma\_brucei | Good | 99 | |
| G5J(S) | Trypanosoma\_brucei | Trypanosoma\_brucei | Good | 99 | |
| G15(G) | Trypanosoma\_brucei | Trypanosoma\_brucei | Good | 99 | |
| G16(B) | Trypanosoma\_brucei | Trypanosoma\_brucei | Fair | 98 | |
| G16J(SA) | Trypanosoma\_brucei | Trypanosoma\_brucei | Good | 95 | |
| G17J (G) | Trypanosoma\_brucei | Trypanosoma\_brucei | Good | 99 | |
B: blood, G: gonadal adipose, H: heart adipose , I: Intestine adipose, O: other adipose, SA: skin adipose, S: skin
